# ACKR3 agonism induces heterodimerization with chemokine receptor CXCR4 and attenuates platelet function

**DOI:** 10.1111/eci.14327

**Published:** 2024-10-07

**Authors:** Valerie Dicenta‐Baunach, Zoi Laspa, David Schaale, Manuel Sigle, Alp Bayrak, Tatsiana Castor, Thanigaimalai Pillaiyar, Stefan Laufer, Meinrad Paul Gawaz, Anne‐Katrin Rohlfing

**Affiliations:** ^1^ Department of Cardiology and Angiology University Hospital Tübingen, Eberhard Karls University Tübingen Tübingen Germany; ^2^ Institute of Pharmaceutical Sciences, Department of Pharmaceutical and Medicinal Chemistry Eberhard Karls University Tübingen Tübingen Germany; ^3^ Tübingen Center for Academic Drug Discovery & Development (TüCAD2) Eberhard Karls University Tübingen Tübingen Germany; ^4^ iFIT Cluster of Excellence EXC 2180 ‘Image‐Guided and Functionally Instructed Tumor Therapies’ Eberhard Karls University Tübingen Tübingen Germany

**Keywords:** atypical chemokine receptor 3, C‐X‐C motif chemokine ligand 12, C‐X‐C motif chemokine receptor type 4, heterodimerization, platelet

## Abstract

**Background:**

Platelet receptors ACKR3 and CXCR4 play a crucial role in a variety of cardiovascular diseases. Like most chemokine receptors, CXCR4 is a G protein coupled receptor that induces platelet activation. In contrast, the atypical chemokine receptor 3 (ACKR3) lacks the ability to activate heterotrimeric G proteins and its activation leads to platelet inhibition and attenuates thrombus formation. In nucleated cells, heterodimerization of ACKR3 with CXCR4 regulates CXCL12‐dependent signalling. The aim of our study was to investigate the formation of ACKR3/CXCR4 heterodimers in platelets and the subsequent consequences for platelet function.

**Methods and Results:**

Using a proximity ligation assay (PLA, Duolink®) to screen for CXCR4/ACKR3 heterodimerization inducing compounds, we found that ACKR3 agonism but not conventional platelet agonists or endogen ligands lead to heterodimer formation. To further characterize the formation of ACKR3/CXCR4 heterodimers, we studied the CXCL12‐dependent platelet activation via CXCR4. Both, CXCL12‐dependent platelet aggregation and collagen‐dependent ex vivo thrombus formation were significantly downregulated by ACKR3 agonism. Moreover, platelet intracellular calcium and Akt signalling were increased by CXCL12 and again suppressed by ACKR3‐specific agonists. Previously, CXCL12 was shown to decrease platelet cAMP levels via CXCR4. Treatment with a specific ACKR3 agonist counteracted this CXCL12/CXCR4‐dependent cAMP decrease.

**Conclusion:**

Our results reveal that the formation of platelet ACKR3/CXCR4 heterodimers is dependent on ACKR3 rather than CXCR4. Furthermore, ACKR3 agonism induced heterodimerization is associated with mitigating CXCL12/CXCR4‐dependent platelet activation possibly by modulating CXCR4‐dependent G protein signalling. Our results indicate possible ACKR3 agonist functions and reinforce the potential therapeutic applications of ACKR3 agonists.

## Graphical abstract

Platelet chemokine receptors play a crucial role in platelet signalling and function. Unlike most other chemokine receptors, the atypical chemokine receptor ACKR3 is a platelet inhibitory receptor. Here, we demonstrate that ACKR3 agonism leads to heterodimerization with chemokine receptor CXCR4 in platelets, thereby inhibiting CXCR4 platelet activation signalling. CXCL12‐dependent platelet aggregation, thrombus formation and signalling via CXCR4 is significantly diminished by ACKR3 agonism and therefore associated with CXCR4/ACKR3 heterodimerization.

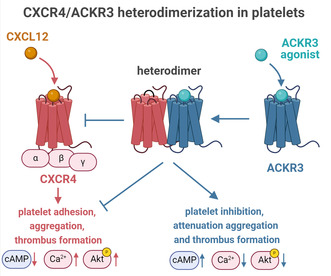

## 1 INTRODUCTION

Chemokine receptors play an important role in platelet function.^1^, ^2^, ^3^, ^4^ Platelets express a variety of chemokine receptors including CCR1 and CCR4, as well as CXCR4, CXCR6 and CX3CR1.^5^, ^6^, ^7^, ^8^ Most chemokine receptors are seven transmembrane‐spanning plasma membrane proteins coupled to heterotrimeric G proteins (GPCRs).^9^, ^10^, ^11^ Interaction of chemokines with their respective chemokine receptors primarily promotes platelet activation.^5^, ^6^, ^7^, ^12^, ^13^, ^14^ In contrast, the atypical chemokine receptor 3 (ACKR3, formerly CXCR7) has been recently identified to attenuate platelet activation.^15^, ^16^, ^17^, ^18^ Binding of macrophage migration inhibitory factor (MIF) to ACKR3 mitigates activation and prevents platelets from undergoing apoptosis.^17^ Further, genetic deficiency of ACKR3 in mice results in hyperreactivity of platelets^15^ and specific ACKR3 agonists inhibit platelet activation and functions.^15^, ^16^, ^19^

Surface expression of ACKR3 is dynamically regulated. Stimulation of platelets with CXCL12 (C‐X‐C motif chemokine ligand 12, also SDF‐1α), an endogen ligand of both CXCR4 and ACKR3, results in downregulation of CXCR4 and upregulation of ACKR3 within the platelet outer plasma membrane.^18^ This dynamic surface expression of CXCR4 and ACKR3 is regulated via ERK1/2 and cyclophilin A signalling.^18^ In nucleated cells, co‐expression of CXCR4 and ACKR3 results in an increased formation of CXCR4/ACKR3 heterodimers and regulates CXCL12‐mediated signalling.^20^, ^21^ Whether CXCR4/ACKR3 heterodimerization occurs in anucleated platelets and affects functions is unknown.

The purpose of our study was to characterize the surface expression and formation of CXCR4/ACKR3 heterodimerization in platelets and to elucidate its consequence for CXCL12‐dependent platelet activation and thrombus formation.

## 2 MATERIALS AND METHODS

### 2.1 Materials

Recombinant human CXCL12 and CXCL14 were purchased from R&D systems (R&D Systems, Minneapolis, Minnesota, USA). ACKR3 agonist VUF11207 was obtained from Merck (Merck KGaA, Darmstadt, Germany). Compound 23 (C23) and control compound C46 were designed and synthesized by ourselves. In our screening, C23 showed promising affinity (EC_50_ = 111 nM), selectivity and high potency to inhibit platelet activation. For a detailed description of the agonist development and characterization please refer to Bayrak et al., 2022 and 2024.^16^, ^22^ Standard chemicals were purchased from Carl Roth (Karlsruhe, Germany) or Merck (Darmstadt, Germany). Platelet agonist CRP‐XL (collagen related peptide) was obtained from CambCol Laboratories (Cambridge, UK), adenosine diphosphate (ADP) from Probe & go Labordiagnostica (Lemgo, Germany) and thrombin from Merck (Merck KGaA, Darmstadt, Germany). Prostaglandin E_1_ (PGE_1_) was purchased from Merck (Merck KGaA, Darmstadt, Germany).

### 2.2 Preparation of human platelets

Prior blood collection, healthy donors gave informed written consent (local ethics committee vote 141/2018B02), the study methodologies conformed to the standards set by the Declaration of Helsinki. Acid‐citrate‐dextrose anticoagulated blood (50 mM citric acid, 85 mM trisodium citrate, pH 4.69, 1:5 (v:v)) was centrifuged at 200 ×*g* for 20 min, and the resulting platelet rich plasma was washed in modified Tyrode's‐HEPES buffer (137 mM NaCl, 2.8 mM KCl, 12 mM NaHCO_3_, 5 mM glucose, 0.4 mM Na_2_HPO_4_, 10 mM HEPES and pH 6.5) without bovine serum albumin by centrifuging again at 830 ×*g* for 10 min. After discarding the supernatant, the platelet pellet was resuspended in Tyrode's‐HEPES buffer (pH 7.4), and the platelet count was determined using a SYSMEX cell counter (Sysmex Cooperation, Kobe, Japan).

### 2.3 Proximity ligation assay

The proximity ligation assay Duolink® (Merck KGaA, Darmstadt, Germany) was performed according to the manufacturer's instructions. 1 × 10^6^ washed platelets for microscopy analysis and 2 × 10^6^ for flow cytometry analysis in Tyrode's‐HEPES buffer (pH 7.4) supplemented with 1 mM CaCl_2_ were treated as indicated and afterwards fixed with 2% formalin. For microscopy, the nonadherent platelets were centrifuged onto coverslips. After every staining and washing step, the flow cytometry samples were centrifuged at 400 ×*g* for 5 min in a V bottom 96 well plate. Both experimental sets were blocked with 10% donkey serum (Merck KGaA, Darmstadt, Germany) and 1% bovine serum albumin (AppliChem, Darmstadt, Germany) in phosphate‐buffered saline. Afterwards, the platelets were incubated at 4°C over night with antibodies against CXCR4 (MAB172, Clone # 44716, R&D Systems, Minneapolis, Minnesota, USA), ACKR3 (ab72100, abcam, Cambridge, United Kingdom) or corresponding IgG controls (Mouse IgG2B Isotype Control MAB004 R&D Systems, Minneapolis, Minnesota, USA and rabbit IgG Isotype Control 3900S Cell Signaling Technology, Danvers, MA, USA). The following PLA steps, binding of the PLA probes (oligonucleotide‐labelled secondary antibodies), ligation, and signal amplifications were performed according to the manufacturer's protocol. For microscopic analysis, the platelets were stained for 30 min with Phalloidin Alexa Fluor® 488 (ThermoFisher Scientific, Waltham, Massachusetts, USA) before mounting. Fluorescence microscopy images were taken on a Nikon Eclipse Ti2‐A microscope (100x DIC objective, Nikon, Tokyo, Japan). The images were analysed with the NIS‐Elements AR software version 5.21 (Nikon, Tokyo, Japan). The PLA pixel count was evaluated in relation to the phalloidin pixel count, which serves as platelet quantification. For flow cytometry measurements, platelets were washed a last time with wash buffer and the platelets were solved in 150 μL PBS after centrifugation. For acquisition the BD FACSLyric™ Flow Cytometry System (BD Bioscience, Franklin Lakes, NJ, USA) was used. Data were analysed using the FlowJo™ Software, the gating strategy is depicted in Figure S1B (version 10.10, BD Bioscience, Franklin Lakes, NJ, USA).

### 2.4 Immunoblot analysis

2.5 × 10^6^ washed platelets in Tyrode's‐HEPES buffer (pH 7.4) supplemented with 1 mM CaCl_2_ were treated as indicated and subsequently lysed using RIPA lysis buffer. The protein amount was measured using a standard Bradford assay. Platelet lysates were electrophoretically separated by sodium dodecyl sulfate‐polyacrylamide gel electrophoresis (SDS–PAGE, 10%) under reducing conditions and transferred onto a polyvinylidene difluoride membrane (PVDF). PVDF membranes were blocked with Roti‐Block (Carl Roth, Karlsruhe, Germany) or 3% bovine serum albumin (Merck KGaA, Darmstadt, Germany) in TRIS‐TWEEN‐buffered saline and incubated overnight with the primary antibodies as indicated. Antibodies against phospho‐Akt (sites T308 and S473) and total Akt were obtained from Cell Signaling Technology (Danvers, MA, USA) and loading control anti‐GAPDH was purchased from ThermoFisher Scientific (Waltham, Massachusetts, USA). IRDye secondary antibodies (Li‐Cor, Lincoln, Nebraska, USA) were used in a 1:15,000 dilution in Roti‐Block or 3% bovine serum albumin/TRIS‐TWEEN‐buffered saline. For fluorescence detection and analysis, the LI‐COR Odyssey System (Li‐Cor, Lincoln, Nebraska, USA) was used.

### 2.5 Platelet aggregometry

Platelet aggregation was measured with citrate‐anticoagulated platelet rich plasma (1 × 10^8^ platelets/ sample) using a light transmission aggregometer (Aggregometer 490‐X; Chrono‐Log Corp., Havertown, Pennsylvania, USA).^23^ Platelets were treated as indicated and aggregation was measured for 5 min with a stir speed of 1,000 rpm at 37°C. The extent of aggregation was quantified in percentage of light transmission and analysed using Aggrolink8 software (Chrono‐Log Corp., Havertown, Pennsylvania, USA).^7^

### 2.6 Ex vivo thrombus formation

For ex vivo thrombus formation experiments, a flow chamber system (Maastricht Instruments B. V., Maastricht, The Netherlands) with collagen‐coated cover slips (100 μg/mL Kollagen Reagens HORM suspension, Takeda, Tokyo, Japan) at a shear rate of 1000 s^−1^ was used. Washed platelets (5 × 10^5^) in Tyrode's‐HEPES buffer (pH 7.4) or CPDA anticoagulated whole blood were diluted 4:5 in phosphate‐buffered saline containing 1.2 mM Ca^2+^. Subsequently, whole blood or platelets were treated as indicated and stained for 10 min with fluorochrome 3,3′‐dihexy‐loxacarbocyanine iodide (DiOC_6_, Sigma Aldrich, St. Louis, Missouri, USA). For visualization, a Nikon fluorescence microscope was used (Nikon Eclipse Ti2‐A, 20x objective, Nikon, Tokyo, Japan) and at least five images of independently selected areas were taken after the perfusion. The images were analysed with the NIS‐Elements AR software version 5.21 (Nikon, Tokyo, Japan).

### 2.7 Calcium signalling

Microscopic platelet intracellular Ca^2+^ was measured as described previously using 5 μM Fluo‐4 fluorescence dye (Invitrogen, Waltham, Massachusetts, USA).^24^ 1 × 10^5^ washed platelets were labelled with Fluo‐4 for 30 min at room temperature (RT), incubated on fibrinogen (100 μg/mL, Sigma Aldrich, St. Louis, Missouri, USA) coated coverslips for 20 min and pretreated with or without 100 μM ACKR3 agonists or control C46 for 15 min at RT. After incubation, nonadherent platelets were removed, fresh Tyrode's‐HEPES buffer (pH 7.4) was added and platelets were analysed using a fluorescence microscope (Nikon Eclipse Ti2‐A; DIC 100× oil objective, Nikon, Tokyo, Japan). Fluo‐4 fluorescence was recorded for 90 s and after 15 s platelets were activated as indicated. To estimate the cytosolic Ca^2+^ activity, the fold change in the mean intensity of single platelet fluorescence was determined using the NIS‐Elements AR software version 5.21 (Nikon, Tokyo, Japan). Additionally, calcium flow (Fluo‐4) was measured using flow cytometry. 1 × 10^6^ washed platelets per sample were preincubated with 2.5 μM Fluo‐4 in 50 μL modified Tyrode's without Ca^2+^ for 30 min at RT. Subsequently, the volume was adjusted to 300 μL and platelets treated as indicated. Before CXCL12 activation, a control measurement was captured. After activation, Fluo‐4 signals were measured for the timepoints 0 min, .5 min and 1 min. For quantification, the area under the curve of the Fluo‐4 over time curves was used.

### 2.8 Measurement of cAMP levels

To determine platelet levels of cyclic adenosine monophosphate (cAMP) an enzyme‐linked immunosorbent assay (ELISA, Enzo Life Sciences, Farmingdale, New York, USA) was used. Platelets were treated as indicated and lysed using a lysis buffer containing .5% TritonX‐100 and 1 mM IBMX (3‐Isobutyl‐1‐methyl‐Xanthin) to stop endogenous phosphodiesterase activity. After centrifugation at 12,000 ×*g* for 5 min at RT, the supernatant was used to perform the ELISA according to the manufacturer's instructions.

### 2.9 Statistical analysis and graphical presentation

Data are provided as means ± SD and *n* represents the number of biological replicates. Repeated measures of one‐way ANOVA or mixed‐effects analysis with an appropriate post‐test, as indicated, were performed for multiple comparisons. Two‐sided Student's *t*‐tests were utilized for two group comparison (*p* < .05 statistical significance, 95% confidence interval) or Wilcoxon matched‐pairs signed rank test for not normally distributed data. All statistical analyzes were performed with GraphPad Prism (GraphPad Software, Inc., La Jolla, CA, USA, Version 10.1.1). Schematic drawings were created using BioRender.com.

## 3 RESULTS

### 3.1 Heterodimers of ACKR3 with CXCR4 occur on the platelets surface upon ACXCR3 agonism

**FIGURE 1:**
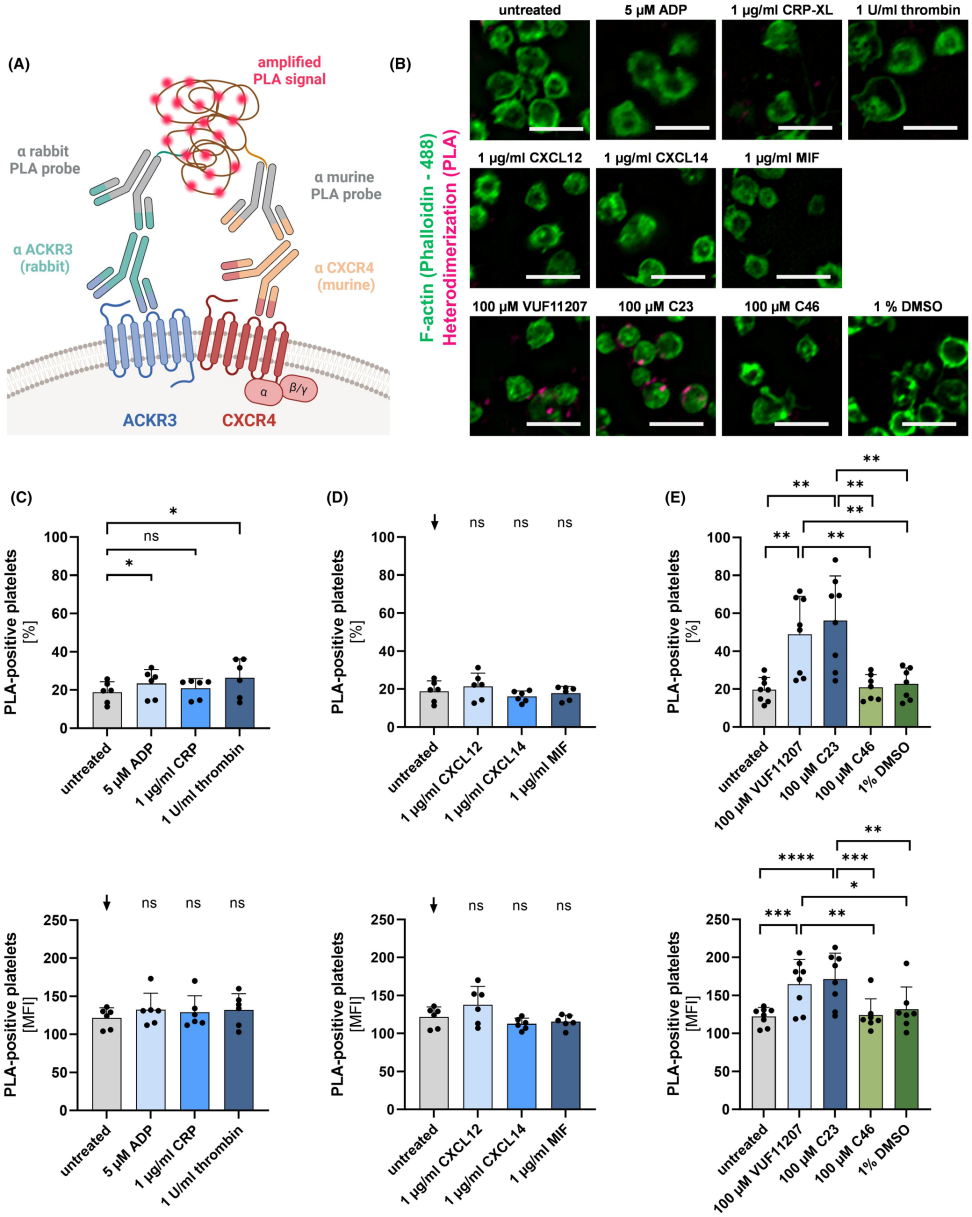
Heterodimers of ACKR3 and CXCR4 occur on platelets surface upon ACXCR3 agonism. (A) Scheme of proximity ligation assay Duolink® functioning. ACKR3 and CXCR4 are labelled with specific primary antibodies of different species. These are detected with the corresponding PLA probes with short reverse DNA tails, which are linked in the ligation step if the two receptors are in close proximity to each other. The signal is amplified by a polymerization reaction. Created with BioRender.com. (B) Representative images of PLA (magenta) and phalloidin (green) staining of untreated platelets or treated platelets with 5 μM ADP, 1 μg/mL CRP‐XL and 1 U/mL thrombin for 30 min at RT (upper panel) or with 1 μg/mL CXCL12, 1 μg/mL CXCL14, 1 μg/mL MIF (middle panel) or 100 μM ACKR3 agonist VUF11207 and C23 and control substance C46/DMSO (lower panel) for 15 min at RT. Scale bar = 5 μm. (C–E) PLA flow cytometry analysis of ACKR3‐CXCR4 interaction. (C) Platelets treated with 5 μM ADP, 1 μg/mL CRP‐XL and 1 U/mL thrombin for 30 min at RT compared to untreated control. Upper graph: Statistical analysis of ACKR3‐CXCR4 PLA positive platelets [%], Plotted: Arithmetic means ± SD of PLA positive platelets [%], *n* = 6, statistics: RM one‐way ANOVA (compared to untreated); n.s. not significant, *p < .05. Lower graph: Statistical analysis of mean fluorescence intensity [MFI] of ACKR3‐CXCR4 PLA positive platelets. Plotted: Arithmetic means ± SD of PLA positive platelets [MFI], *n* = 6, statistics: RM one‐way ANOVA (black arrow: Compared to untreated); n.s. not significant. (D) Platelets treated with 1 μg/mL CXCL12, 1 μg/mL CXCL14, 1 μg/mL MIF for 15 min at RT compared to untreated control. Upper graph: Statistical analysis of ACKR3‐CXCR4 PLA positive platelets [%], Plotted: Arithmetic means ± SD of PLA positive platelets [%], *n* = 6, statistics: RM one‐way ANOVA (black arrow: Compared to untreated); n.s. not significant. Lower graph: Statistical analysis of mean fluorescence intensity [MFI] of ACKR3‐CXCR4 PLA positive platelets, Plotted: Arithmetic means ± SD of PLA positive platelets [MFI], *n* = 6, statistics: RM one‐way ANOVA (black arrow: Compared to untreated); n.s. not significant. (E) Platelets treated with 100 μM ACKR3 agonist VUF11207 and C23 and control substance C46 /DMSO for 15 min at RT compared to untreated control. Upper graph: Statistical analysis of ACKR3‐CXCR4 PLA positive platelets [%], Plotted: Arithmetic means ± SD of PLA positive platelets [%], *n* ≥ 7, statistics: RM one‐way ANOVA; **p < .01. Lower graph: Statistical analysis of mean fluorescence intensity [MFI] of ACKR3‐CXCR4 PLA positive platelets. Plotted: Arithmetic means ± SD of PLA positive platelets [MFI], n ≥ 7, statistics: RM one‐way ANOVA; *p < .05, **p < .01, ***p < .001, ****p < .0001.

Platelets dynamically surface express both ACKR3 and CXCR4 upon activation and in response to CXCL12.^18^, ^25^ To explore heterodimerization of both receptors we developed a Duolink® proximity ligation assay (PLA) using a combination of specific anti‐ACKR3 and anti‐CXCR4 antibodies or matching IgG controls (Figure 1A, Figure S1A,B). PLA allows quantifying steric interaction of membrane receptors using oligonucleotide‐labelled secondary antibodies.^26^, ^27^, ^28^ Platelets were stimulated with agonists such as collagen‐related peptide (1 μg/mL CRP‐XL), adenosine diphosphate (5 μM ADP) or thrombin (1 U/mL). Upon activation of platelets with these classical agonists, we did not observe CXCR4/ACKR3 heterodimerization in PLA microscopy (Figure 1B and Figure S1C) as well as flow cytometry experiments (Figure 1C). Previously, we demonstrated that the CXCR4 chemokine CXCL12 in high concentrations decreases the expression levels of CXCR4 and enhances surface exposure of ACKR3.^18^ Thus, we asked whether CXCL12, CXCR4 ligand CXCL14 or ACKR3 ligand MIF induce heterodimerization of both chemokine receptors. As shown in Figure 1B,D and Figure S1C CXCL12 and CXCL14 did not stimulate CXCR4/ACKR3 heterodimerization. Next, we asked, whether ACKR3 agonists promote interaction of CXCR4 with ACKR3. For this study, we used the commercially available ACKR3 agonist VUF11207 and the newly developed ACKR3 agonist compound 23 (C23).^22^ C23 displayed high activity, selectivity and potency as well as low toxicity.^22^ Interestingly, we found that the ACKR3‐selective agonists significantly enhanced formation of CXCR4/ACKR3 heterodimers on the platelet surface shown in flow cytometry experiments (Figure 1E) as well as microscopy analysis (Figure 1B and Figure S1C). At the same time, ACKR3 agonists reduce the overall receptor presentation of ACKR3 and CXCR4 on the platelet surface (Figure S1D,E), whereas endogen ligands did not altered the surface expression and classical activators did not affect the expression or even significantly increased the receptor expression (Figure S1D,E). Thus, we conclude that heterodimer formation between ACKR3 and CXCR4 occurs on the platelet surface specifically following ACKR3 agonism and may precede receptor internalization.

### 3.2 ACKR3 agonists attenuate CXCL12‐dependent platelet aggregation and thrombus formation under flow

**FIGURE 2:**
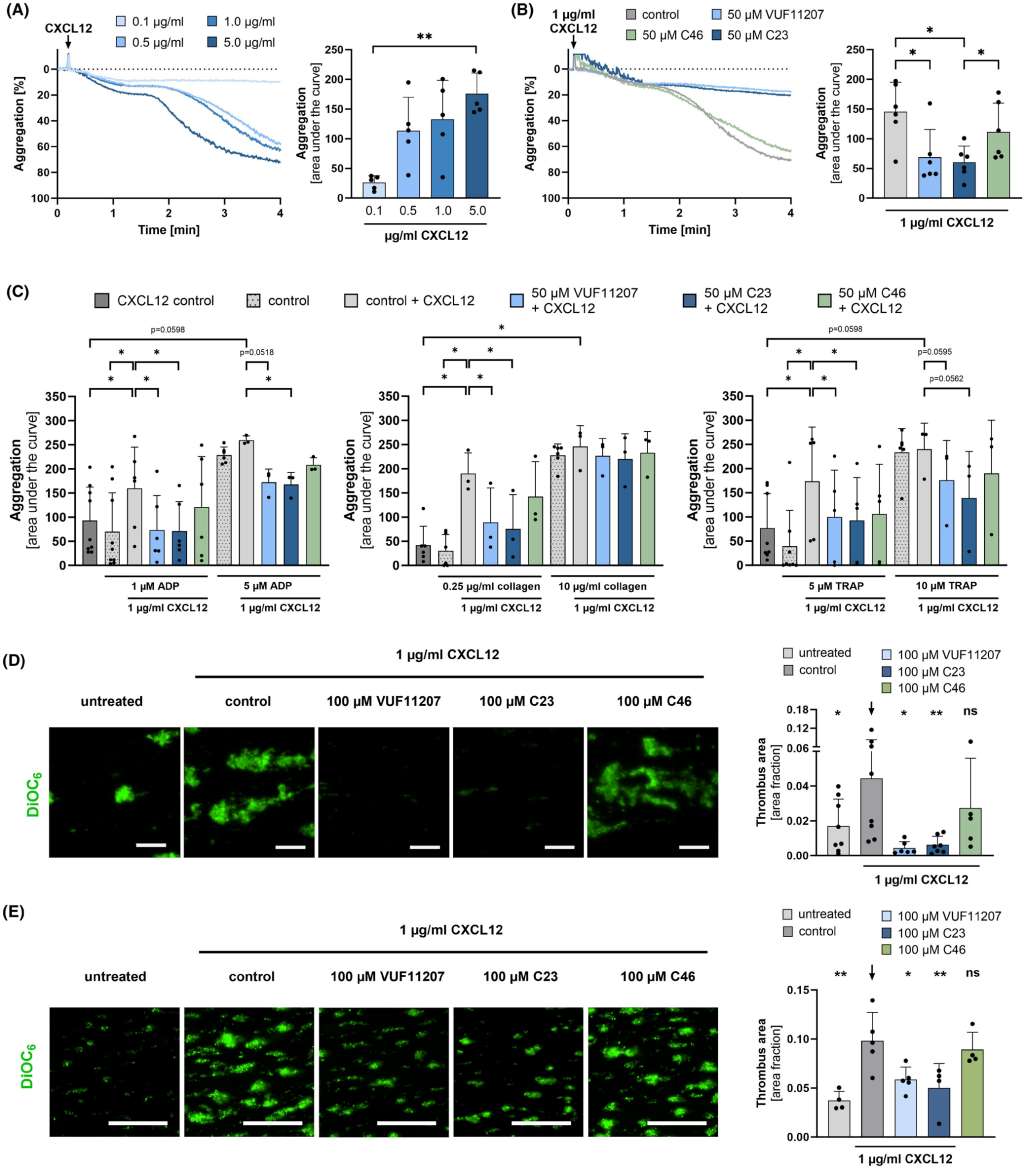
ACKR3 agonist‐induced heterodimerization of ACKR3/CXCR4 is associated with attenuation of CXCL12‐dependent platelet function. (A) Representative light transmission aggregation curves (left) and statistical analysis (right) of platelets treated with .1, .5, 1.0 and 5.0 μg/mL CXCL12. Plotted: Arithmetic means ± SD of platelet aggregation [%], *n* = 5, statistics: RM one‐way ANOVA, not indicated = n.s. not significant, **p < .01. (B) Representative light transmission aggregation curves (left) and statistical analysis (right) of platelets pretreated with 50 μM ACKR3 agonist (VUF11207 or C23) or 50 μM control substance C46 for 15 min at 37°C and activated with 1.0 μg/mL CXCL12. Plotted: Arithmetic means ± SD of platelet aggregation, *n* = 6, statistics: RM one‐way ANOVA, not indicated = n.s. not significant, *p < .05. (C) Statistical analysis of CXCL12‐dependent light transmission aggregometry in combination with threshold and high concentrations of ADP (left), collagen (middle), and TRAP (right). Plotted: Arithmetic means ± SD of platelet aggregation [%], *n* ≥ 3, statistics: RM one‐way ANOVA; not indicated = n.s. not significant, *p < .05. (D, E) Representative microscope images of DiOC_6_ stained platelets (left, scale bar 100 μm) and statistical analysis (right) of ex vivo thrombus formation. D Washed platelets or E whole blood were pretreated with 100 μM ACKR3 agonist (VUF11207 or C23) or 100 μM control substance C46 for 15 min at RT and activated with 1.0 μg/mL CXCL12 for 10 min. Plotted: Arithmetic means ± SD of thrombus area fraction, *n* ≥ 4, statistics: Wilcoxon matched‐pairs signed rank test for not normally distributed data (black arrow: Compared to CXCL12 control); n.s. not significant, *p < .05, **p < .01.

CXCL12 induces platelet activation and aggregation via ligation of CXCL12 with CXCR4.^5^, ^12^, ^14^, ^29^ In our present study, we confirm that CXCL12 stimulates platelet aggregation in a dose‐dependent manner (Figure 2A). To test whether ACKR3‐agonist‐induced CXCR4/ACKR3 receptor heterodimerization is associated with platelet function, isolated human platelets were stimulated with recombinant CXCL12 (1 μg/mL) and the platelet response was analysed by light transmission aggregometry in the presence and absence of ACKR3 agonists (Figure 2B). We found that in the presence of ACKR3 agonists (VUF11207, C23) but not a control chemical (C46), CXCL12‐induced platelet aggregation was significantly inhibited (control vs. VUF11207: *p* < .05 and control vs. C23: *p* < .05) (Figure 2B). In addition, we tested the effect of ACKR3 agonists on the synergistic‐induction of platelet aggregation by CXCL12 and classical platelet activators (ADP, collagen, TRAP). At threshold concentrations of the classical activators (1 μM ADP, .25 μg/mL collagen, 5 μM TRAP), each in combination with 1 μg/mL CXCL12, we detected a significant inhibition of aggregation (Figure 2C). Whereas high activator concentrations (5 μM ADP, 10 μg/mL collagen and 10 μM TRAP) in conjecture with 1 μg/mL CXCL12 did not increase the solely with the classical activators induced aggregation. Further, both ACKR3 agonists had just partial or no effect on this high synergistic platelet aggregation (Figure 2C). Next, we tested the effect of ACKR3 agonism on CXCL12‐dependent platelet thrombus formation on immobilized collagen under flow. As described for aggregation, CXCL12 increased in vitro thrombus formation of washed platelets as well in whole blood samples (Figure 2D,E). Platelet‐dependent thrombus formation in response to CXCL12 was substantially reduced in the presence of ACKR3 agonists VUF11207 and C23 but not the control chemical C46 (Figure 2D,E). Our data imply that ACKR3 agonist‐dependent CXCR4/ACKR3 heterodimerization is associated with a reduction of platelet function in response to CXCL12.

### 3.3 CXCL12‐dependent platelet signalling is inhibited by ACKR3 agonism

**FIGURE 3:**
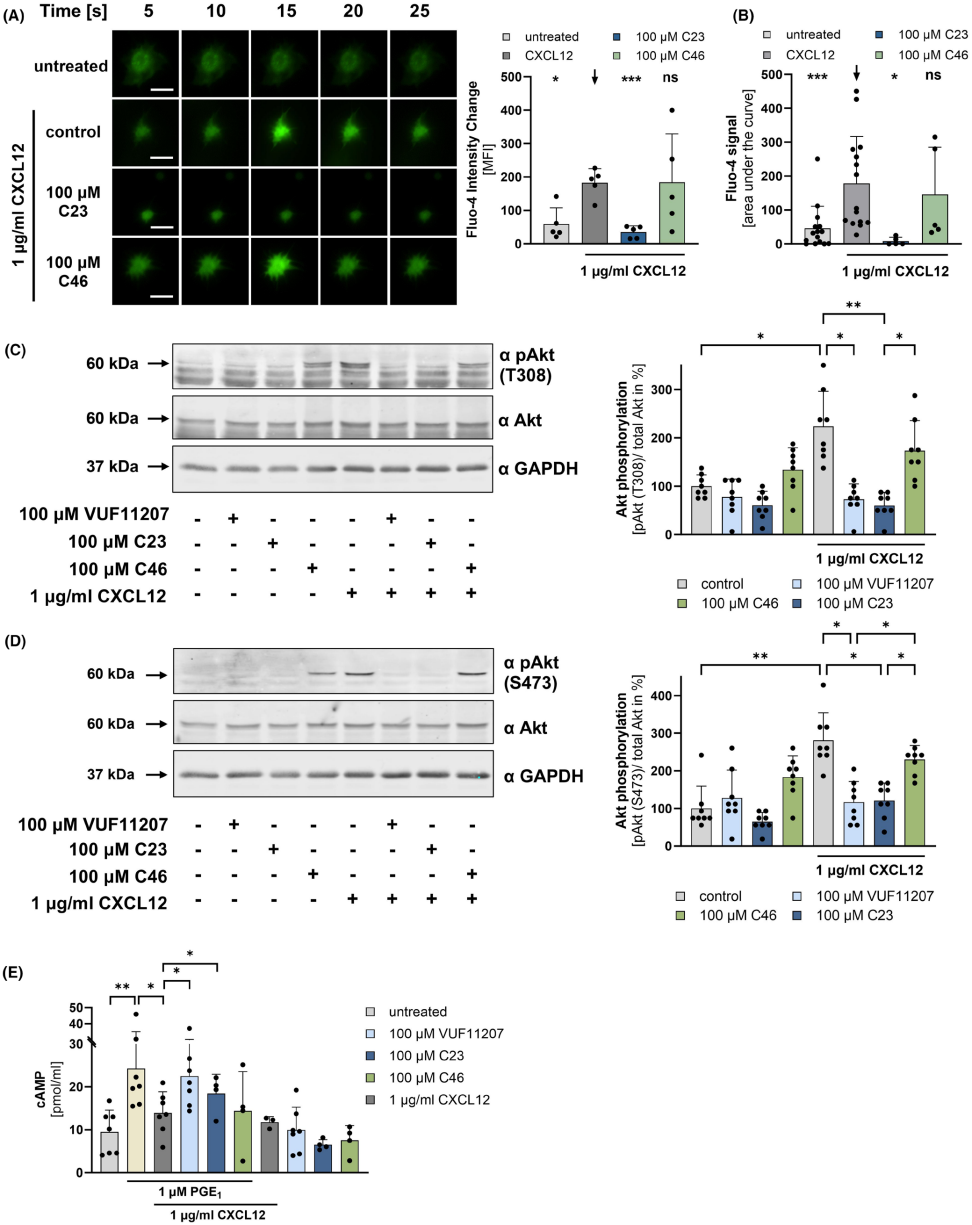
CXCL12‐dependent platelet signalling is inhibited by ACKR3 agonism. (A, B) Intracellular calcium signalling of platelets using Fluo‐4 staining (30 min RT). (A) Representative microscope images (left, scale bar 5 μm) and statistical analysis (right) of washed platelets pretreated with 100 μM ACKR3 agonist C23 or 100 μM control substance C46 for 15 min at RT and activated with 1.0 μg/mL CXCL12. Plotted: Arithmetic means ± SD of Fluo‐4 intensity change, *n* = 5, ordinary one‐way ANOVA (black arrow: Compared to CXCL12 control); n.s. not significant, *p < ß.05, ***p < .001. (B) Statistical analysis of flow cytometry measurements of washed platelets pretreated with 100 μM ACKR3 agonist C23 or 100 μM control substance C46 for 15 min at RT and activated with 1.0 μg/mL CXCL12. Plotted: Arithmetic means ± SD of Fluo‐4 MFI signal, *n* ≥ 5, ordinary one‐way ANOVA (black arrow: Compared to CXCL12 control); n.s. not significant, *p < .05, ***p < .001. (C, D) Representative images of western blots detecting Akt phosphorylation at (C) T308 as well as (D) S473 of washed platelets pretreated with or without 100 μM ACKR3 agonists (VUF11207, C23) or control substance (C46) and subsequent platelet activation with 1 μg/mL CXCL12 and statistical analysis of the densitometric measurements of the AKT phosphorylation signals. Plotted: Arithmetic means ± SD of pAkt/Akt in percent, *n* = 8, ordinary one‐way ANOVA Dunnett's multiple comparisons test; not indicated = n.s. not significant, *p < .05, **p < .01. (E) cAMP levels of washed platelets pretreated with or without 100 μM ACKR3 agonist VUF11207 and C23 as well as control substance C46 for 15 min at RT and subsequent platelet treatment with 1 μM PGE_1_ and 1 μg/mL CXCL12 for 10 min at 37°C. Plotted: Arithmetic means ± SD of cAMP concentration, *n* = 7 (CXCL12: *N* = 3; C23 and C46: *N* = 4), student's *t*‐test; not indicated = n.s. not significant, *p < .05, **p < .01.

Recently, we described that ACKR3 ligation modulates platelet activation signalling.^15^, ^30^ To test the effect of ACKR3 agonism on CXCL12‐dependent Ca^2+^ signalling, platelets were loaded with the fluorescent calcium indicator Fluo‐4 and treated with CXCL12 (1 μg/mL). CXCL12 substantially induced an increase in intracellular Ca^2+^ measured by microscopy techniques with platelets settled on fibrinogen (Figure 3A). Single cell analysis by flow cytometry confirmed the microscopy data (Figure 3B). CXCL12‐induced Ca^2+^ signalling was significantly attenuated in the presence of ACKR3 agonist C23 compared to controls (Figure 3A,B). Further, CXCL12‐induced Akt phosphorylation in a concentration‐dependent manner (data not shown). As described above for intracellular Ca^2+^ signalling, CXCL12‐dependent Akt phosphorylation at threonine 308 as well as serine 473, and consequently Akt activation, was significantly reduced in the presence of ACKR3 agonist VUF11207 and C23 (*T308*: CXCL12 vs. CXCL12 + VUF11207: *p* < .05; CXCL12 vs. CXCL12 + C23: *p* < .001; *S473*: CXCL12 vs. CXCL12 + VUF11207: *p* < .05; CXCL12 vs. CXCL12 + C23: *p* < .05) (Figure 3C,D). Previously, CXCL12 has been shown to initiate platelet thrombus formation through CXCR4 and changes in cAMP and Ca^2+^ signalling.^14^ Furthermore, cAMP has been shown to inhibit Akt phosphorylation and thereby AKT‐mediated signalling.^31^, ^32^ Thus, we asked whether ACKR3 agonism counteracts CXCL12‐dependent cAMP suppression. We used prostaglandin E_1_ (PGE_1_) to stimulate cAMP expression in platelets. Indeed, we were able to demonstrate that CXCL12 significantly reduced PGE_1_‐induced cAMP expression. Furthermore, we detected that CXCL12‐dependent suppression of the cAMP concentration is partially reversed in the presence of ACKR3 agonists VUF11207 and C23 (CXCL12 vs. CXCL12 + VUF11207: *p* < .05; CXCL12 vs. CXCL12 + C23: *p* < .05) (Figure 3E).

**FIGURE 4:**
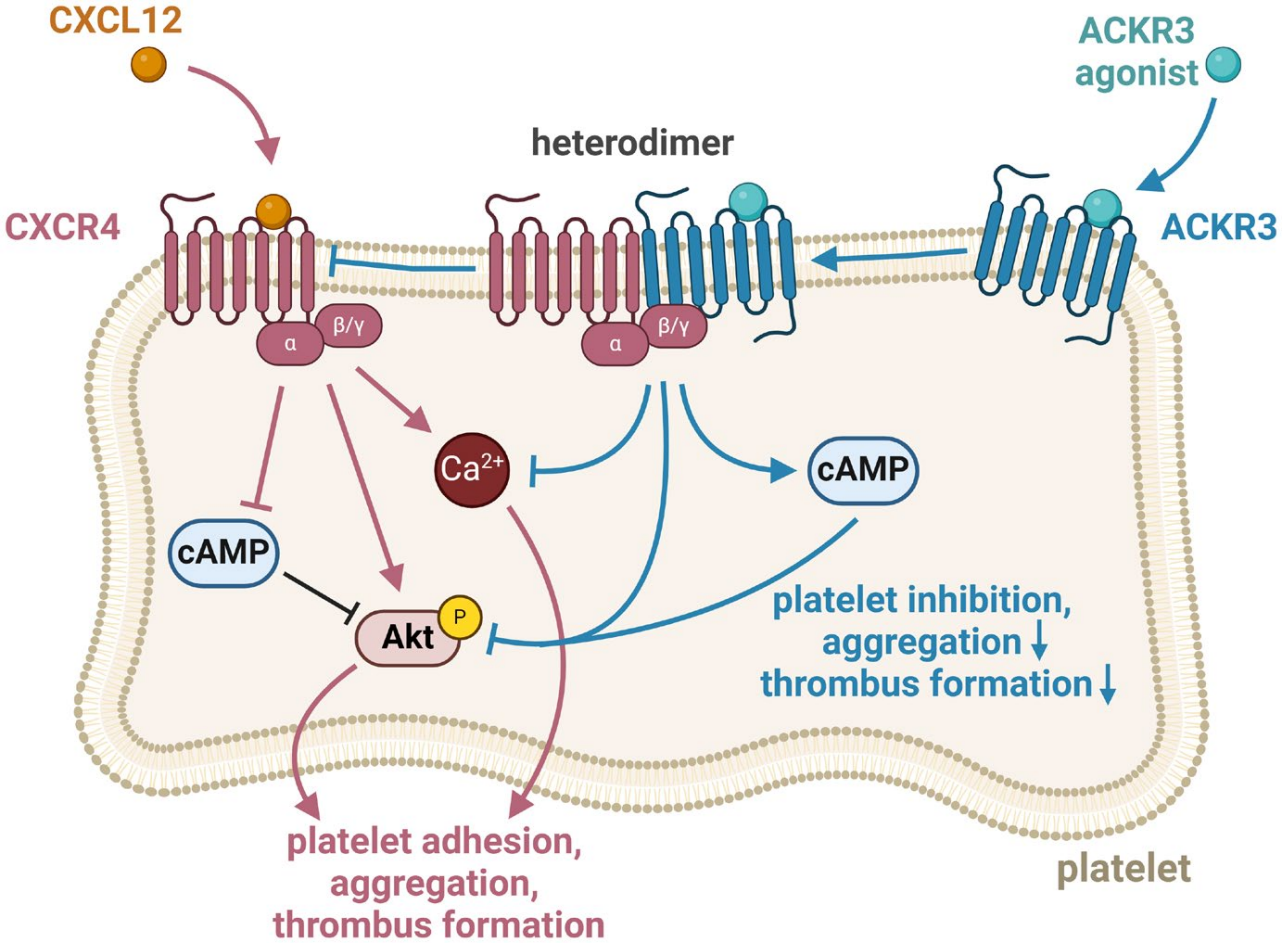
CXCR4/ACKR3 heterodimer associated signalling in platelets. Schematic drawing of CXCR12 dependent‐signalling via CXCR4. ACKR3 agonism induced CXCR4/ACKR3 heterodimerization mitigates CXCL12/CXCR4‐dependent platelet adhesion, aggregation and thrombus formation by counteracting cAMP inhibition and attenuation of Akt and Ca^2+^ signalling. Created with BioRender.com.

In summary, ACKR3‐induced heterodimerization of CXCR4/ACKR3 is associated with significant inhibition of platelet aggregation and thrombus formation. A function that is regulated via Ca^2+^‐cAMP‐Akt signalling (Figure 4). Our data imply that heterodimerization of ACKR3 with CXCR4 modulates the responsiveness of CXCR4 towards its ligand CXCL12.

## 4 DISCUSSION

The major findings of the present study are the following: (i) Selective ACKR3 agonism induces ACKR3 heterodimerization with CXCR4 (ii) ACKR3 agonists attenuate CXCL12‐dependent platelet aggregation and thrombus formation. (iii) CXCL12‐dependent platelet signalling is mitigated by ACKR3 agonism.

Unlike most other chemokine receptors, ACKR3 is a platelet inhibitory receptor.^15^, ^17^ The endogenous CXCR4 and ACKR3 ligand MIF inhibits platelet activation and apoptosis via ACKR3.^17^ Recently, it has been shown that patients suffering from coronary artery disease with elevated ACKR3 levels had an improved clinical prognosis and platelets showed less aggregation.^15^, ^30^ The murine platelet‐specific ACKR3 knockout exhibits hyperreactive platelets, leading to increased ex vivo thrombus formation and platelet activation as well as degranulation.^15^ In addition, animals carrying this platelet specific *Ackr3* knockout showed increased damage and inflammation of the myocardium and brain after ischemia/reperfusion.^15^ Targeting ACKR3 has been shown to inhibit platelet activation.^16^ Previously, the use of ACKR3 agonists has been shown to inhibit human and murine platelet activation and aggregation and to increase cAMP levels and the amount of antiplatelet lipids.^30^ In addition, in murine in vivo experiments, agonists were able to reduce thrombo‐inflammation and infarct size without affecting basal haemostasis.^15^, ^30^ Those findings encouraged us to investigate the role of ACKR3 agonism in CXCR4/ACKR3 heterodimerization and platelet function. In the present study, we show that the formation of platelet CXCR4/ACKR3 heterodimers is dependent on ACKR3 agonism. ACKR3 agonism, but not platelet agonists (such as ADP or collagen‐related peptide) nor the CXCR4 ligands CXCL12 and CXCL14 or the ACKR3 ligand MIF significantly increased the PLA signal of CXCR4/ACKR3 heterodimers. To get further insights in the role of CXCR4/ACKR3 heterodimers for platelets, we investigated CXCL12‐dependent platelet function and signalling. CXCL12 is the endogenous ligand of ACKR3 and CXCR4, whereby the binding to the latter in platelets leads to internalization and subsequent ACKR3 externalization.^18^, ^33^ CXCL12 is secreted, inter alia, by platelets and is therefore a paracrine and autocrine platelet agonist^12^, ^34^ especially potentiating other platelet agonists such as ADP and fibrinogen.^5^, ^35^, ^36^ These findings are in line with our aggregation and thrombus formation experiments. CXCL12 induced concentration‐dependent platelet aggregation and increased the thrombus area onto immobilized collagen under flow. Both, CXCL12‐dependent aggregation and thrombus size were significantly attenuated using specific ACKR3 agonists. Even CXCL12‐dependent aggregation aggravated with different classical platelets agonists was decreased using ACKR3 agonists. Previously, CXCL12 was shown to induce platelet aggregation via CXCR4 rather than ACKR3.^14^, ^29^ Together with our findings, this indicates that CXCR4/ACKR3 heterodimerization is associated with the inhibition of CXCL12‐dependent platelet activation via CXCR4. Unlike ACKR3, which lacks the ability to activate heterotrimeric G proteins, CXCR4 is a G protein coupled receptor (GPCR). Analog to the ADP receptor P2Y_12_, CXCR4 is coupled to G_αi_ and CXCL12 ligation leads to G_αi_‐dependent inhibition of the adenylyl cyclase (AC).^12^, ^14^ Thus, the level of platelet modulating cAMP is decreased. Indeed, PGE_1_‐induced platelet cAMP levels were reduced using CXCL12 and again, ACKR3 agonism counteracted this effect. Further, CXCL12‐dependent Akt and calcium signalling was diminished using a selective ACKR3 agonist. Lou et al. (2002) and Makhoul et al. (2019) demonstrated that cAMP is able to inhibit Akt phosphorylation.^31^, ^32^ Our data indicate that AC‐cAMP signalling is modulated by CXCL12 ligation to CXCR4. Furthermore, by CXCL12‐dependent reduction of the cAMP levels, Akt phosphorylation and intracellular calcium are increased, which is counteracted by ACKR3 agonism. It was shown that heterodimerization of CXCR4 and ACKR3 in HEK293T cells changed the ability of CXCR4 to interact with its G proteins.^21^ Thus, it is conceivable that ACKR3 agonism leads to CXCR4/ACKR3 heterodimerization, possibly followed by internalization, which is associated with the modulation of CXCL12‐dependent interaction of CXCR4 with its coupled heterotrimeric G proteins.

Platelet ACKR3 and CXCR4 play a crucial role in a variety of cardiovascular diseases^15^, ^25^, ^37^ and it was recently shown that platelet‐specific CXCL12 knockout mice also show limited arterial thrombosis without prolonging the bleeding time.^38^ Thus, this expands the possible use of specific ACKR3 agonists for the therapeutic regulation of platelets.

## AUTHOR CONTRIBUTIONS

V.D.‐B., M.P.G. and A.‐K.R. conceived and designed research. V.D.‐B., Z.L., D.S., M.S. and A.B., performed experiments and analysed data. V.D.‐B., Z.L., D.S., M.S., M.P.G. and A.‐K.R. interpreted results of experiments. V.D.‐B., M.P.G. and A.‐K.R. prepared figures and drafted manuscript. Z.L., D.S., M.S., A.B., T.C., T.P., S.L., M.P.G. and A.‐K.R. edited and revised manuscript. All authors have read and agreed to the published version of the manuscript.

## CONFLICT OF INTEREST STATEMENT

No conflicts of interest, financial or otherwise, are declared by the authors.

## Supporting information


Figure S1.

## Data Availability

For original data please contact anne-katrin.rohlfing@med.uni-tuebingen.de.
