# Supplementary material for: ACKR3 agonism induces heterodimerization with chemokine receptor CXCR4 and attenuates platelet function
Source: Eur J Clin Invest. 2024 Oct 7;55(1):e14327. doi: 10.1111/eci.14327 (PMC11628653; doi:10.1111/eci.14327)
Supplement: Supplementary file 1 — Figure S1. [file ECI-55-e14327-s001.docx]

**Supplementary material**

**ACKR3 agonism induces heterodimerization with chemokine receptor CXCR4 and attenuates platelet function**

Valerie Dicenta-Baunach^1^ M.Sc., Zoi Laspa^1^ RPh, David Schaale^1^, Manuel Sigle^1^ PhD, Alp Bayrak^2^ M.Sc., Tatsiana Castor^1^ PhD, Thanigaimalai Pillaiyar^2,3^ PhD, Stefan Laufer^2,3,4^ PhD, Meinrad Paul Gawaz^1^ PhD, Anne-Katrin Rohlfing^1^ PhD

^1^Department of Cardiology and Angiology, University Hospital Tübingen, Eberhard Karls University Tübingen, Tübingen, Germany; ^2^Institute of Pharmaceutical Sciences, Department of Pharmaceutical and Medicinal Chemistry, Eberhard Karls University Tübingen, Tübingen, Germany; ^3^Tübingen Center for Academic Drug Discovery & Development (TüCAD2), Eberhard Karls University Tübingen, Tübingen, Germany; ^4^iFIT Cluster of Excellence EXC 2180 ‘Image-Guided and Functionally Instructed Tumor Therapies’, Eberhard Karls University Tübingen, Tübingen, Germany

**Corresponding author**

Anne-Katrin Rohlfing, PhD

Department of Cardiology and Angiology, University Hospital Tübingen

Eberhard Karls University Tübingen

Otfried-Müller-Str. 10; 72076 Tübingen, Germany

Tel.: +49 7071 29 82887; Fax: +49 7071 29 4474

E-mail: [anne-katrin.rohlfing@med.uni-tuebingen.de](mailto:anne-katrin.rohlfing@med.uni-tuebingen.de)

**Supplementary Figure 1**

**Supplementary Figure 1.** **ACKR3 and CXCR4 presentation on the platelets surface upon ACXCR3 agonism. A** Representative microscopic images of PLA (magenta) and phalloidin (green) staining of **left** IgG control staining and **right** receptor specific antibodies for the experiments depicted in **Figure 1B**. Scale bar = 5 µm. **B** Gating strategy for the flow cytometry analysis of the CXCR4-ACKR3 PLA depicted in **Figure 1C-E**. **C** Statistical analysis of the microscopic images of PLA samples as presented in **Figure 1B** for ACKR3-CXCR4 heterodimerization, **left** platelet activation for 30 min with 5 µM ADP, 1 ug/ml CRP-XL and 1 U/ml thrombin compared to untreated control **middle** platelet treatment with 1 µg/ml CXCL12, 1 µg/ml CXCL14 or MIF **right** platelet treatment with 100 µM ACKR3 agonists (VUF11207, C23), 100 µM control substance C46 and vehicle control 1% DMSO. Plotted: arithmetic means ± SD of PLA signal per platelet signal. Left: platelet agonists, n≥4, statistics: Wilcoxon matched-pairs signed rank test for not normally distributed data; n.s. not significant. Middle: CXCR4/ACKR3 ligands, n=6 (n=3 for MIF), statistics: RM one-way ANOVA (compared to untreated, black arrow); n.s. not significant. Right: ACKR3 agonists and respective controls, n≥7, statistics: Wilcoxon matched-pairs signed rank test for not normally distributed data; *p < 0.05. **D** Flow cytometric measurements of the CXCR4 surface expression under various treatments. **left** platelet activation for 30 min with 5 µM ADP, 1 ug/ml CRP-XL and 1 U/ml thrombin compared to untreated control **middle** platelet treatment with 1 µg/ml CXCL12 or 1 µg/ml CXCL14 **right** platelet treatment with 100 µM ACKR3 agonists (VUF11207, C23), 100 µM control substance C46 and vehicle control 1% DMSO. Plotted: arithmetic means ± SD of CXCR4 mean fluorescence, n=3, statistics: RM one-way ANOVA (black arrow: compared to untreated); n.s. not significant, *p<0.05. **E** Flow cytometric measurements of the ACKR3 surface expression under various treatments. **left** platelet activation for 30 min with 5 µM ADP, 1 ug/ml CRP-XL and 1 U/ml thrombin compared to untreated control **middle** platelet treatment with 1 µg/ml CXCL12 or 1 µg/ml CXCL14 **right** platelet treatment with 100 µM ACKR3 agonists (VUF11207, C23), 100 µM control substance C46 and vehicle control 1% DMSO. Plotted: arithmetic means ± SD of ACKR3 mean fluorescence, n=3, statistics: RM one-way ANOVA (black arrow: compared to untreated); n.s. not significant, *p<0.05.
